# Quantum correlation dynamics subjected to critical spin environment with short-range anisotropic interaction

**DOI:** 10.1038/srep32634

**Published:** 2016-09-06

**Authors:** J. L. Guo, X. Z. Zhang

**Affiliations:** 1 College of Physics and Materials Science, Tianjin Normal University, Tianjin 300387, China; 2 Tianjin International Joint Research Centre of Surface Technology for Energy Storage Materials, Tianjin Normal University, Tianjin 300387, China

## Abstract

Short-range interaction among the spins can not only results in the rich phase diagram but also brings about fascinating phenomenon both in the contexts of quantum computing and information. In this paper, we investigate the quantum correlation of the system coupled to a surrounding environment with short-range anisotropic interaction. It is shown that the decay of quantum correlation of the central spins measured by pairwise entanglement and quantum discord can serve as a signature of quantum phase transition. In addition, we study the decoherence factor of the system when the environment is in the vicinity of the phase transition point. In the strong coupling regime, the decay of the decoherence factor exhibits Gaussian envelop in the time domain. However, in weak coupling limit, the quantum correlation of the system is robust against the disturbance of the magnetic field through optimal control of the anisotropic short-range interaction strength. Based on this, the effects of the short-range anisotropic interaction on the sudden transition from classical to quantum decoherence are also presented.

The quantum aspects of correlations in composite systems are a key issue in quantum information theory^1^. Quantum entanglement, which determines the given state is separable or not, has been regarded as a valuable resource for quantum information processing^2^. Even many people take it granted that quantum entanglement is quantum correlation. However, some separate states also contains quantum correlation and there exist quantum tasks that display the quantum advantage without entanglement^3^, so entanglement is not the only type of quantum correlation. Quantum discord (QD) defined as the difference between quantum mutual information and classical correlation^4^, is supposed to characterize all of nonclassical correlations including entanglement. Such states with non-zero QD but not entanglement may be responsible for the efficiency of a quantum computer^5^,^6^. Consequently, QD is believed a new resource for quantum computation.

Meanwhile, study of quantum phase transition (QPT)^7^ purely driven by quantum fluctuations can help us understand the physical properties of various matters from the perspective of quantum mechanics. During the past decade, the central spin model served as a paradimatic model characterizing the interaction between the quantum system and surrounding environment has received a lot of attentions^8^,^9^,^10^. On the one hand, it can provide a platform to investigate the underlying mechanism of the decoherence^11^,^12^ due to the exact solvability of the model, which can pave the way to develop new methods that enhance the coherence time in the context of quantum computation and information^1^,^13^. On the other hand, one can identify the quantum phase transition through the quantum-classical transition of the system described by a reduction from a pure state to a mixture^14^. This stimulates a series of works regarding the disentanglement of central spins subjected to critical surrounding environment^10^,^15^,^16^,^17^,^18^,^19^,^20^,^21^. It was shown that at the critical point where the environment occurs QPT, the decoherence is enhanced, and the disentanglement process is accelerated by the quantum criticality. Recently, QD was analyzed in this context^22^,^23^,^24^. The results show that the quantum discord is more robust than entanglement for the system exposed to the spin environment, and a signature of the QPT can be available through the QD even when the entanglement is absent.

In general, the surrounding system possessing the short-range interaction is more closer to the real spin environment comparing to the standard one with only nearest-neighbor couplings. Recently, Zhang *et al*.^25^ propose a class of exactly solvable Ising models including short-range anisotropic interaction. These models can exhibit rich phase diagrams, which correspond to various geometric shapes in the auxiliary space. In addition, the geometric topology of these models ensures that the corresponding ground states are robust with respect to the variation of the system parameters in some extent. Motivated by this discovery, we investigate the dynamical quantum correlation of two-qubit system coupled to the XY spin chain with short-range anisotropic interaction. We find that the decay of the quantum correlation of the system measured by entanglement and QD can be deemed as a signature to characterize the quantum phase transition of the surrounding environment. On the other hand, counter-intuitively, we show that the introduction of the anisotropic interaction will not change the critical point of the environment but can suppress the decoherence of the system in the weak coupling regime, which can provide the possibility to prepare the states with long coherence time in the experimental demonstration. Based on this, we also study the effect of the anisotropic interaction on the sudden transition from classical to quantum decoherence.

## Results

### Hamiltonian evolution

The total Hamiltonian for two central qubits coupled to an XY spin chain with three-site anisotropic interaction we considered in this paper is described by


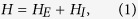


where


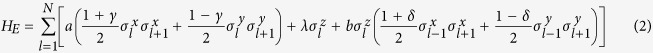


denotes the Hamiltonian of the environmental spin chain, and


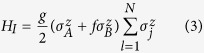


is the interaction Hamiltonian between the two-qubit system and the spin chain. 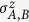 and 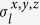 are the Pauli operators used to describe the two qubits and the environmental spin chain respectively. *N* is the number of spins in the chain and the periodic boundary conditions are satisfied. *λ* represents the strength of the transverse field applied to the spin chain and *b* denotes the three-site interaction. *γ* and *δ* describe the anisotropy of the system arising from the nearest-neighbor qubits and the next-nearest-neighbor qubits respectively. In the case of *δ* = 0, *H*_*E*_ reduces to the XY spin chain with isotropic three-site interaction^26^. *g* is coupling strength between the two-qubit system and the spin chain. The parameter *f* ∈ (0, 1) denotes the two qubits couple asymmetrically to the spin chain. *f* = 0 indicates only one spin of the two qubits is coupled with the spin chain and *f*  = 1 indicates the two qubits are coupled together with the same spin chain. Notice that 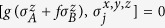, the total Hamiltonian can be rewritten as


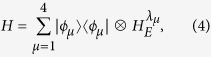


where |*ϕ*_*μ*_〉 are the *μ*th eigenstate of the operator 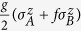 and read |*ee*〉, |*eg*〉, *ge*〉, |*gg*〉 corresponding to the *μ*th eigenvalue *g*_*u*_. The parameters *λ*_*μ*_ are given by *λ*_*μ*_ = *λ* + *g*_*μ*_ taking the following expressions 
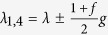
, 
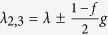
. Then 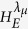 can be obtained from *H*_*E*_ by replacing *λ* with *λ*_*μ*_.

Let’s assume that the two-qubit system and the environmental spin chain are initially in the product density matrix form


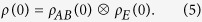


Where *ρ*_*AB*_(0) and 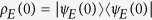 are the initial density matrixes of the two-qubit system and the environmental spin chain. Then the time evolution of the total system is governed by 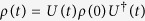 with *U*(*t*) = exp(−*iHt*). In order to obtain analytical expression of *U*(*t*), we first need to diagonalize the Hamiltonian 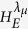. Following the Jordan-Wigner transformation which changes the spin system into a quasi Fermi system


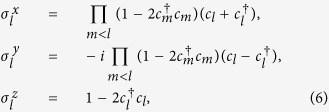


where *c*_*l*_ and 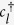 are annihilation and creation operators of spinless fermion. After a straightforward derivation, 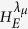 can be written as


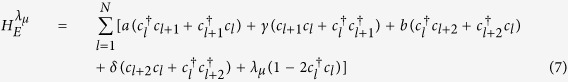


with boundary terms ignored. Then, employing Fourier transforms of the fermionic operators described by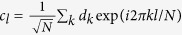, with *k* = −*M*, …, *M* and *M* = (*N* − 1)/2 for odd *N* and Bogoliubov transformation 
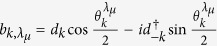
, 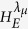 can be diagonalized exactly as


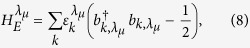


where the energy spectrum is


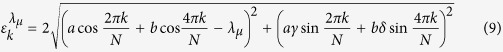


with angles 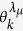 satisfying


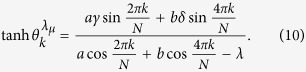


Now we assume that the two-qubit system is initially prepared in the Bell diagonal state


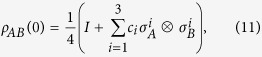


where *c*_*i*_ (0 ≤ |*c*_*i*_| ≤ 1) are the real numbers and *I* is the identity operator. Then the reduced density matrix of two qubits is obtained by tracing out the environment


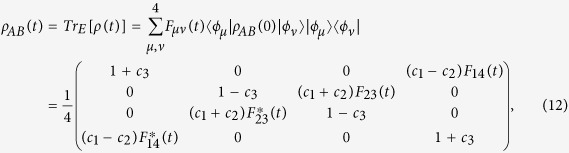


with 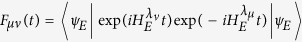. In this work, we assume that the initial state of the environmental spin chain |*ψ*_*E*_(0)〉 is the ground state |*G*〉_*λ*_ of the pure spin-chain Hamiltonian 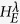. |*G*〉_*λ*_ is the vacuum of the fermionic modes described by *b*_*k*,*λ*_|*G*〉_*λ*_ = 0 and can be written as 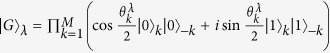, where |0〉_*k*_ and |1〉_*k*_ denote the vacuum and single excitation of the *k*th mode *d*_*k*_, respectively. By using the transformation


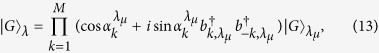


with 
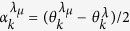
, we can obtain the decoherence factor


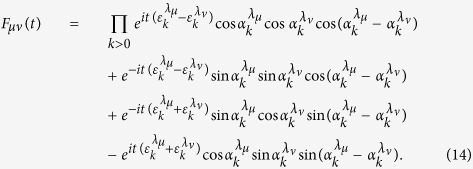


Therefore, we have obtained the reduced density matrix *ρ*_*AB*_(*t*) via which the evolution of quantum correlation for the two-qubit system can be analyzed.

### Concurrence and quantum discord

We now briefly review the definitions of the pairwise entanglement and QD. It is well known that the concurrence defined by Wootters^27^ is a widely accepted measure of entanglement associated with a two-qubit state *ρ*_*AB*_. It can be expressed by *C*(*ρ*_*AB*_) = max{*λ*_1_ − *λ*_2_ − *λ*_3_ − *λ*_4_, 0}, where the quantities *λ*_1_ ≥ *λ*_2_ ≥ *λ*_3_ ≥ *λ*_4_ are the square roots of the eigenvalues of the matrix 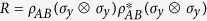. The concurrence *C* = 0 corresponds to a separate state and *C* = 1 for a maximally entangled state. However, as point out above, entanglement is not the only kind of quantum correlation. In quantum information theory, the total correlations between two subsystems *A* and *B* of a bipartite system *ρ*_*AB*_ can be quantified by quantum mutual information *I*(*ρ*_*AB*_) = *S*(*ρ*_*A*_) + *S*(*ρ*_*B*_) − *S*(*ρ*_*AB*_) with *S*(*ρ*) = −*trρ* log *ρ* being von Neumann entropy. While the classical correlation is given by 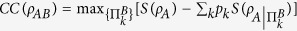 where the maximum is taken over the set of von Neumann measurements 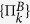 on subsystem *B* and 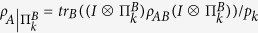 with 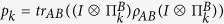. The QD is defined as the difference between the total correlations *I*(*ρ*_*AB*_) and the classical correlation *CC*(*ρ*_*AB*_)^4^, namely


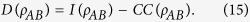


Usually, it is sufficient for us to evaluate QD using the following set of projectors: 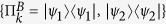, in which 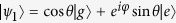 and 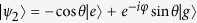 with the parameters *θ* and *φ* varying from 0 to 2*π*. We can obtain the quantum discord via numerical optimization over the parameters *θ* and *φ*. QD can quantify all of the quantum correlation, since it is zero only for state with classical correlation and nonzero for states with quantum correlation.

It is noted that the density matrix (12) has an X-form, and the considered quantum correlation measures for this type of state can be calculated analytically. The concurrence as an entanglement measure is given by


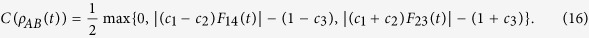


In order to determine quantum correlation measured by QD, we first need evaluate the mutual information, which can be obtained as


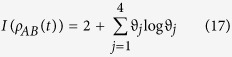


with 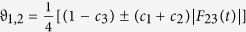 and 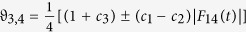. Following the complete set of von Neumann measurement of subsystem A, the classical correlation can be derived as


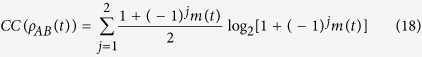


with 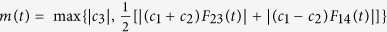. Consequently, we can obtain the expression of QD by eq. (15).

### The evolution of entanglement and quantum discord

First, we consider the case that the two qubits couple with the spin chain equally, i. e. *f* = 1. To start with, we assume that the two qubits are initially in the Bell state 
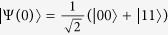
, which corresponds to the state (11) when *c*_1_ = *c*_3_ = 1 and *c*_2_ = −1. According to the definitions of concurrence and QD, we can obtain *C*(*ρ*_*AB*_(*t*)) = |*F*_14_(*t*)| and


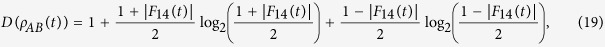


which is shown in detail in Methods section. We can easily find that both the concurrence and QD only involve with |*F*_14_(*t*)| which can be written as


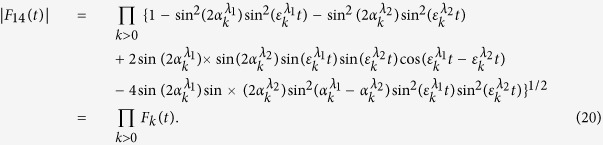


**Figure 1:**
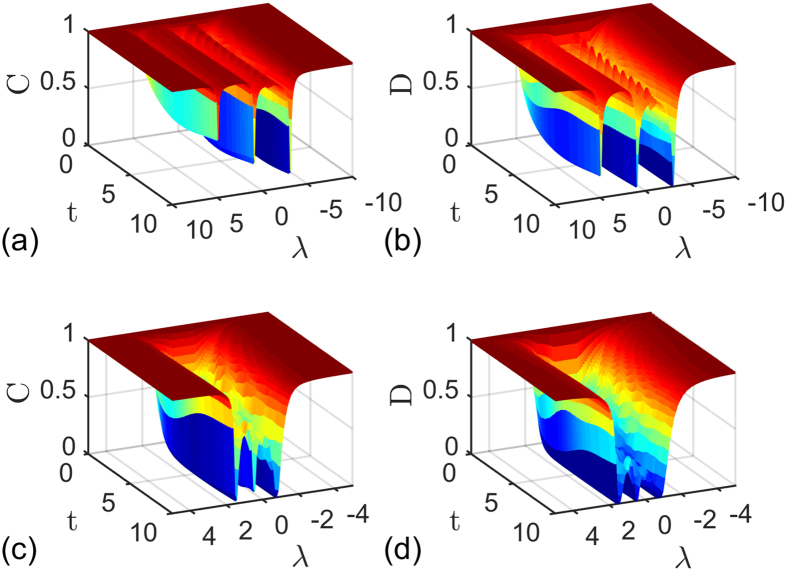
Concurrence (**a**,**c**) and quantum discord (**b**,**d**) as a function magnetic intensity *λ* and time *t* for two qubits prepared in state 
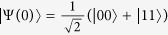
 with parameters (**a**,**b**) *a* = 2, *b* = 3, *δ* = 1 and (**c**,**d**) *a* = 1/2, *b* = 1, *δ* = −1. The other parameters are set to *γ* = 1 and *N* = 1001.

In Fig. 1, the entanglement and QD are plotted as a function of magnetic intensity *λ* and time *t* with different parameters *a*, *b* and *δ*. One can observe that at the critical points *λ*_*c*_ = *a* + *b* = 5, *λ*_*c*_ = *b* − *a* = 1 and *λ*_*c*_ = −*b* = −3 under *a* = 2, *b* = 3, and *δ* = 1, and at the critical points *λ*_*c*_ = *a* + *b* = 3/2, *λ*_*c*_ = *b* − *a* = 1/2 and *λ*_*c*_ = *a*^2^/*b* − *b* = −3/4 under *a* = 1/2, *b* = 1, and *δ* = −1, the entanglement and QD decay more sharply as expected. To understand this effect, taking the case of *a* = 2, *b* = 3, and *δ* = *γ* = 1 as an example, we may turn to the approximation of |*F*_14_(*t*)| given in ref. 10. Here we define a critical value of *k*_*c*_ that corresponds to the critical point of QPT, then noticing that


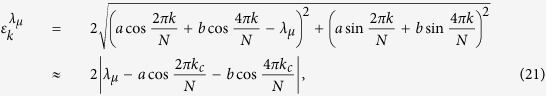


where we keep 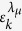 to the zero order of *k* − *k*_*c*_ and use the relation 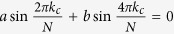 at the critical point of QPT, we have 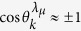 and 
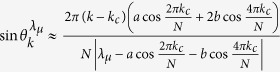
, where we keep 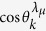 and 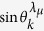 to the zero and first order of *k* − *k*_*c*_, respectively. Simultaneously,


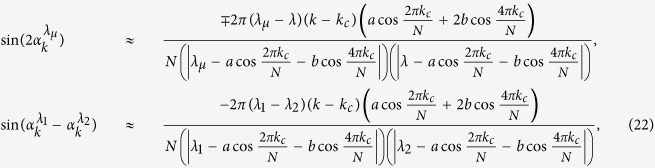


Following the similar procedure of ref. 10, one can introduce a cutoff number *K*_*c*_ and define the partial product for |*F*_14_(*t*)|,


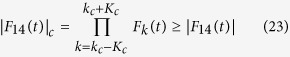


from which the corresponding partial sum is obtained as


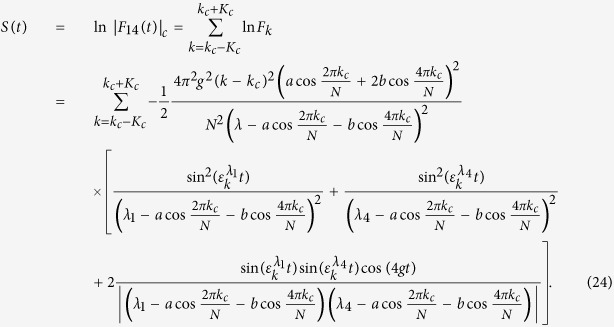


**Figure 2:**
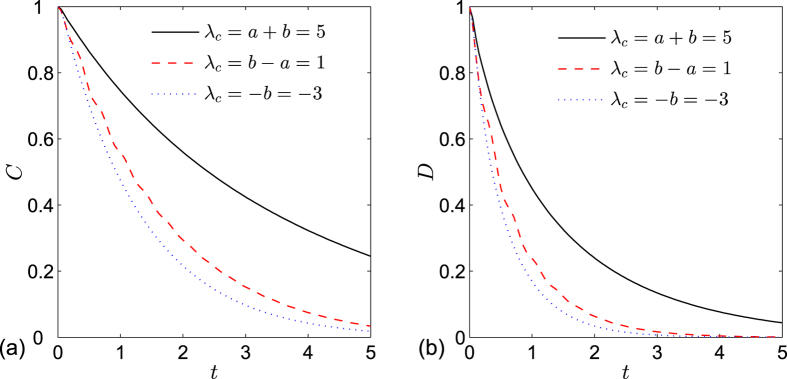
(**a**) Concurrence and (**b**) quantum discord versus time *t* at different critical points for the two qubits prepared in state 
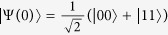
. The other parameters are set to *a* = 2, *b* = 3, *γ* = 1, *δ* = 1, *g* = 0.05 and *N* = 1001.

It is easy to check that 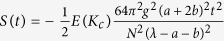 corresponding to *k*_*c*_ = 0, 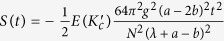 corresponding to 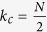, and 
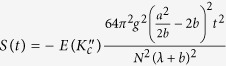
 corresponding to 
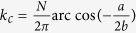
, which indicates that when *λ* → *λ*_*c*_ = *a* + *b*, *b* − *a*, −*b*, |*F*_14_(*t*)| will exponentially decay with the second power of time. In the derivation of the above equation, we employ the approximation 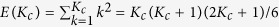, where the value of *K*_*c*_ depends on the parameters related with the spin environment. In order to compare the evolutions of entanglement and QD at different critical points, we give the plot of Fig. 2 from which we can see that these two measures exhibit similar asymptotical decays with respect to time. QD always decays more rapidly than entanglement at the same critical point, and both entanglement and QD at the critical point *λ*_*c*_ = −*b* decay more rapidly than other critical points.

**Figure 3:**
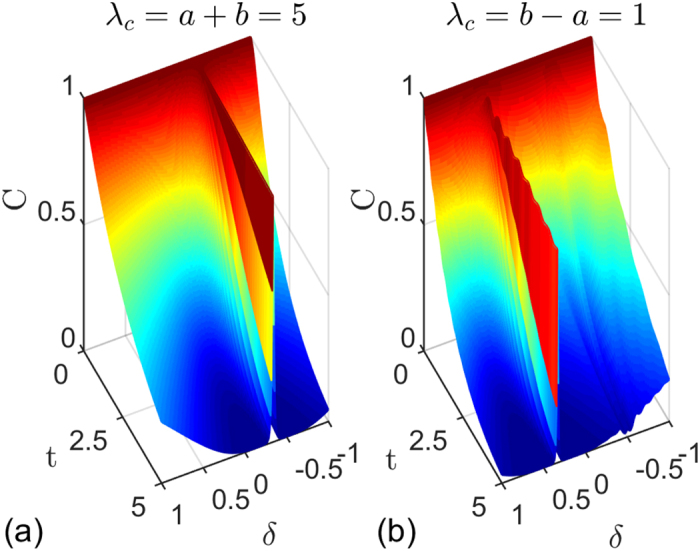
Concurrence as a function of anisotropic parameter *δ* and time *t* at critical points (**a**) *λ*_*c*_ = *a* + *b* and (**b**) *λ*_*c*_ = *b* − *a* for two qubits prepared in state 
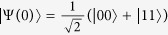
. The other parameters are set to *a* = 2, *b* = 3, *γ* = 1, *g* = 0.05 and *N* = 1001.

Now we consider the effect of anisotropic parameter *δ* on the decays of entanglement and QD. Using detailed numerical calculations we find that the effects induced by *δ* are actually similar to each other on entanglement and QD, so we only give the results of entanglement as a function of anisotropic parameter *δ* and time *t* at critical points in Fig. 3. One can observe that the decay of entanglement can be suppressed with increasing *δ*, though there are some slight oscillations of entanglement with *δ* varying from 0 to −1 at critical point *λ*_*c*_ = *b* − *a*. The most interesting is that when we set 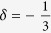 for the case of *λ*_*c*_ = *b* + *a* = 5 and 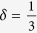 for the case *λ*_*c*_ = *b* − *a* = 1, the entanglement nearly does not change with time. This also can be seen from the expressions of partial sum *S*(*t*) involved with *δ*. For the cases *k*_*c*_ = 0 and *k*_*c*_ = *N*/2, *S*(*t*) has the form 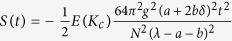 and 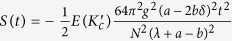 respectively. It is easy to see that when 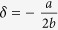 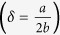, *S*(*t*) → 0 for the case *k*_*c*_ = 0 (*k*_*c*_ = *N*/2), which results in *F*_14_(*t*) → 1. In this sense, we can say that the initial entangled state will immune from the decoherence induced by the spin environment and can be called as a decoherence-free quantum state. Therefore, anisotropy arising from the next-nearest-neighbor qubits can strengthen the quantum correlation between the two qubits and even not results in quantum decoherence in the whole time evolution.

**Figure 4:**
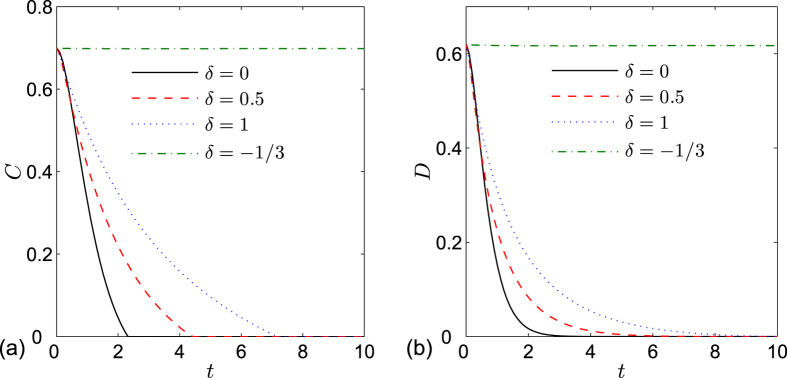
(**a**) Concurrence and (**b**) quantum discord versus time *t* for different values of anisotropic parameter *δ* at critical point *λ*_*c*_ = 5 for two qubits prepared in Werner state with *c* = 0.8. The other parameters are set to *a* = 2, *b* = 3, *γ* = 1, *g* = 0.05 and *N* = 1001.

On the other hand, when the two-qubit system are initially in the mixed state, such as the two-qubit Werner state, which corresponds to the state (11) with *c*_1_ = −*c*_2_ = *c*_3_ = *c* and *c* ∈ [0, 1]. Then we can easily obtain *C*(*ρ*_*AB*_(*t*)) = max[*c*|*F*_14_(*t*)| − (1 − *c*)/2, 0] and 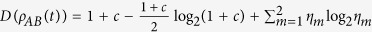 with 
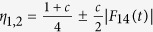
, from which we can clearly see that the entanglement suffers sudden death^28^ and the QD always decays with time asymptotically. This is well known as a unconventional behavior for QD from entanglement. Meanwhile, we notice that the abrupt disappearance of entanglement is harmful for most quantum information processes based on entanglement, so how to suppress this phenomenon is a meaningful work. In Fig. 4, the dynamics of entanglement and QD for different values of anisotropic parameter *δ* at critical point when the two qubits are prepared in Werner state are plotted. We find the death time for entanglement can be delayed and the decay of QD can be released with increasing *δ*, especially when *δ* = −1/3 the phenomenon of entanglement sudden death can be eliminated completely.

**Figure 5:**
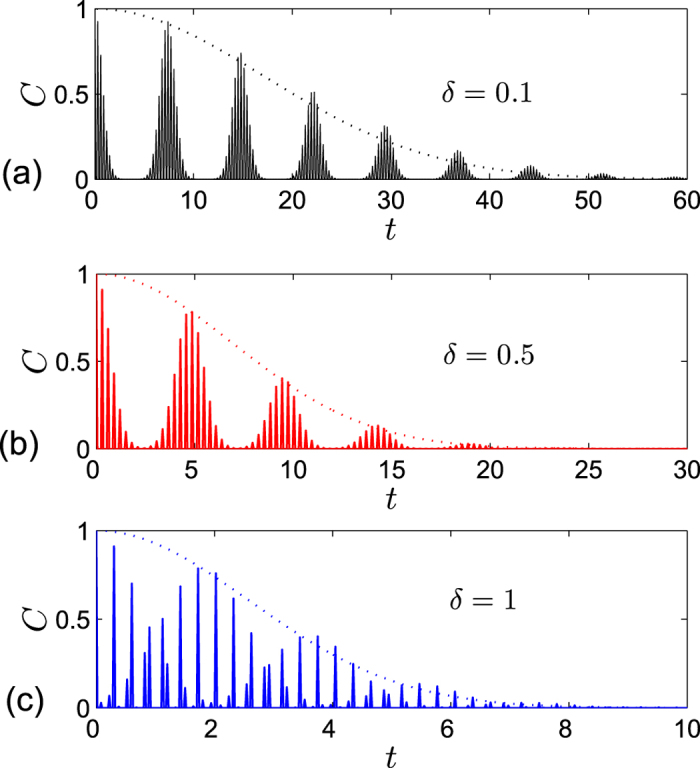
Concurrence (solid line) and approximate Gaussian envelope factor 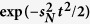 (dotted line) versus time *t* for different values of anisotropic parameter *δ* at critical point *λ*_*c*_ = 5 for two qubits prepared in state 
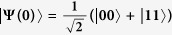
 in strong coupling region. The other parameters are set to *a* = 2, *b* = 3, *γ* = 1, *g* = 500 and *N* = 1001.

In the above discussion we mainly focus on the dynamics of quantum correlation of the two qubits in the weak coupling regime 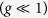. In the following, we will turn to study the case in the strong coupling regime 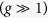. Figure 5 shows the disentanglement process at the critical point for *g* = 500 for the two qubits prepared in Bell state initially. Similar with the results in ref. 10, we find the decay of entanglement is characterized by an oscillatory Gaussian envelop. It is interesting to note that the width of the Gaussian envelop is very sensitive to the anisotropic parameter *δ*. Increasing *δ* will enhance the decay of entanglement, which is in marked contrast to the case in the weak coupling regime where the decay is suppressed as *δ* increases. In fact, from the angle of Bogoliubov transformation one can obtain 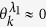, 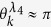, and 
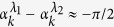
 when 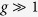, then we have


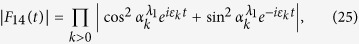


where 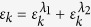. Following the mathematical procedure given in ref. 29, we can obtain


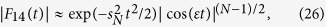


where *ε* is the mean value of *ε*_*k*_ and can be expressed by 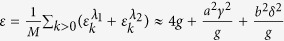. 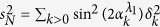, where *δ*_*k*_ describes the derivation of *ε*_*k*_ from its mean values. Its value is 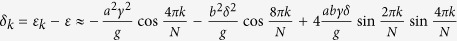, so one can see that the width of the Gaussian envelope is proportional to 
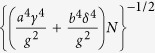
, which is consistent with our numerical results shown in Fig. 5.

**Figure 6:**
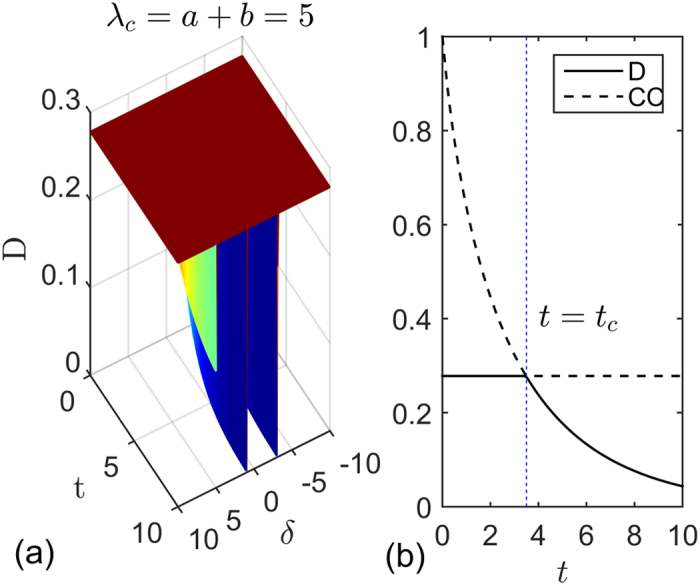
(**a**) Quantum discord as a function of magnetic intensity *λ* and time *t*, and (**b**) quantum discord (solid line) and classical correlations (dashed line) versus time *t* at critical point *λ*_*c*_ = 5, when the two qubits are prepared in Bell diagonal state with *c*_1_ = 1 and *c*_3_ = −*c*_2_ = 0.6. The other parameters are set to *a* = 2, *b* = 3, *δ* = 1, *γ* = 1 and *N* = 2001.

**Figure 7:**
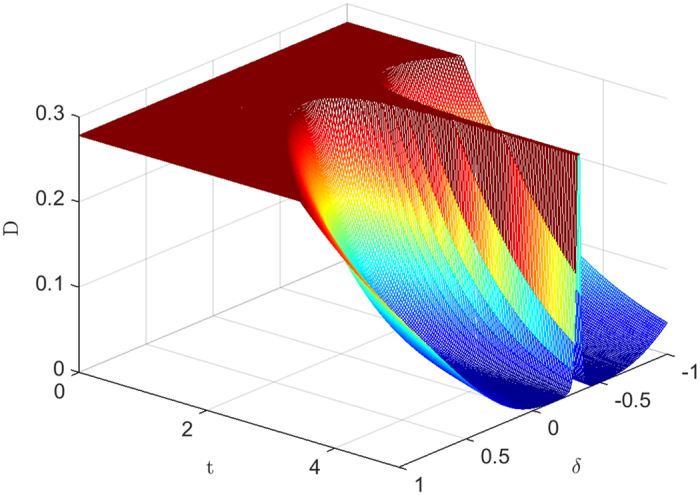
Quantum discord as a function of anisotropic parameter *δ* and time *t* at critical point *λ*_*c*_ = 5 for two qubits prepared in Bell diagonal state with *c*_1_ = 1 and *c*_3_ = −*c*_2_ = 0.6. The other parameters are set to *a* = 2, *b* = 3, *γ* = 1, *g* = 0.05 and *N* = 2001.

Finally, in this section we pay attention to the special case that only one qubit interacts with the spin environment, i.e. *f* = 0. For the initial state with state parameters *c*_1_ = 1, *c*_2_ = −*c*_3_ and |*c*_3_| < 1, it is straightforward to prove


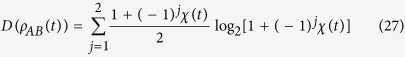


with 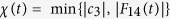, since *F*_14_(*t*) = *F*_23_(*t*) when *f* = 0. Then from ref. 30, we know that there exists a sudden transition from classical to quantum decoherence. As Fig. 6(b) illustrated, classical correlation decreases exponentially and QD retains constant before *t* = *t*_*c*_, after which classical correlation remains constant and QD starts to decrease. However, from Fig. 6(a) we can see that the sudden transition phenomenon only occurs at the critical points of QPT. This implies that the critical points of QPT can also be detected by this phenomenon. In addition, from what has been discussed above, we find that anisotropic parameter *δ* can be regarded as an effective tool to suppress decoherence in weak coupling regime. So one may wonder how will *δ* affect the phenomenon of sudden transition. As is predicated, Fig. 7 shows that the transition time of QD is prolonged as *δ* increases. Therefore, in virtue of anisotropic parameter, we may control the time over which the quantum correlation does not remain constant, which makes it possible to realize quantum computation tasks without any disturbance from the noisy environment for long enough intervals of time.

**Figure 8:**
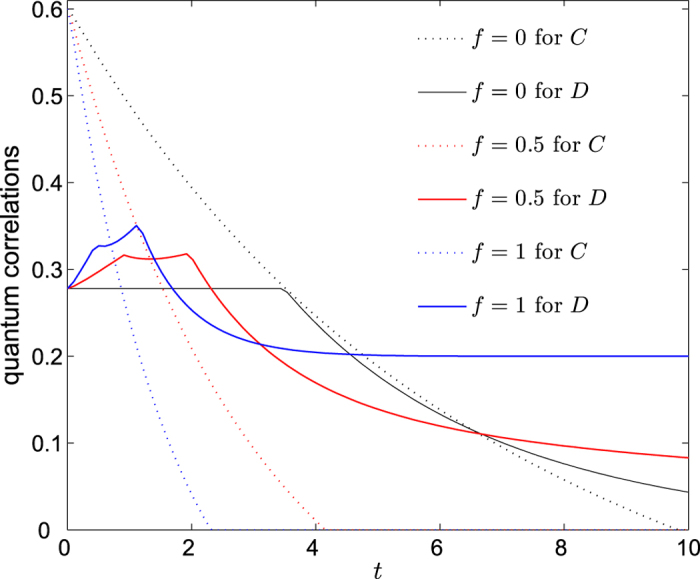
Concurrence and quantum discord versus time *t* at critical point *λ*_*c*_ = 5 for different values of *f*, when the two qubits are prepared in Bell diagonal state with *c*_1_ = 1 and *c*_3_ = −*c*_2_ = 0.6. The other parameters are set to *a* = 2, *b* = 3, *δ* = 1, *γ* = 1 and *N* = 2001.

However, when *f* varies from 0 to 1, we find from Fig. 8 that the phenomenon of sudden transition disappears in the evolution of QD, since the stable regions are replaced by the curves that increase at first and then decrease monotonously to a stable value. The larger the value of *f* is, the greater the stable value of QD reaches. This can be understood by the fact that the two qubits coupled to the same environment, which then in turn generates some effective interaction that strengths the quantum correlation between the two qubits. But we should note that this effective interaction only induce QD, since the entanglement suffers sudden death even more seriously as *f* increases. This once again proves that QD and entanglement are different measures of quantum correlations, and they may behave differently or even contrarily under the same conditions.

## Discussion

In summary, we have investigated quantum correlation of the system coupled to a spin environment with short-range anisotropic interaction. The quantum critical behavior of the surrounding environment can be witnessed by the measures of the entanglement and quantum discord regarding the system. The competition between the magnetic field and short-range anisotropic interaction of the surrounding environment can lead to two distinguishable dynamical behaviors of the two-qubit system. In the weak coupling limit, we have shown that the coherence time can be enhanced through optimal control of the short-range anisotropic interaction even at the quantum phase transition point of the environment, which is robust with respect to the magnetic field. On the contrary, in the strong coupling limit, the decay of the decoherence time presents the Gaussian-like envelop. Furthermore, the effects of the short-range anisotropic interaction on the sudden transition from classical to quantum decoherence are also explored. These findings reveal the effect of the short-range anisotropic interaction on the decoherence of the system, which can pave a new way to prepare the quantum states with long coherence time in real physical realization.

## Methods

To obtain the quantum discord of *ρ*_*AB*_(*t*), i.e.,


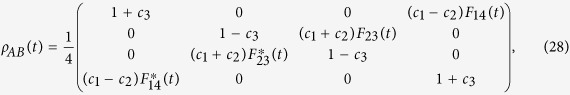


we need to calculate the quantum mutual information and classical correlation. The eigenvalues of the reduced density matrix *ρ*_*AB*_(*t*) can be derived as


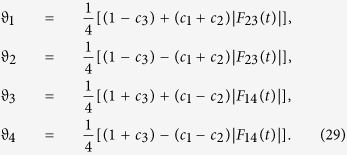


It is not difficult to find from Eq. (28) that *ρ*_*AB*_(*t*) is maximally mixed, which means that *ρ*_*A*_(*t*) = *ρ*_*B*_(*t*) = *I*/2. Consequently, the von Neumann entropy *S*(*ρ*_*A*_(*t*)) = *S*(*ρ*_*B*_(*t*)) = 1. Then, the quantum mutual information between the qubits is


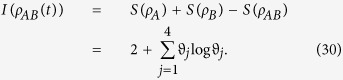


In order to calculate the classical correlation *CC*(*ρ*_*AB*_(*t*)), we choose the complete set of projectors 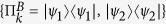 to measure the subsystem *B*, where the two orthogonal projectors are defined by


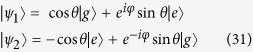


with the parameters *θ* and *φ* varying from 0 to 2*π*. Thus we obtain the reduced density matrices of subsystem *A* after measurement


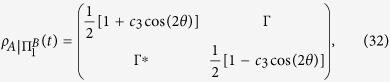



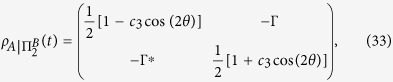


and the probability *p*_1_ = *p*_2_ = 1/2, where


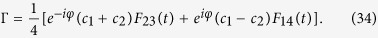


Subsequently the classical correlation of Eq. (28) can be calculated as


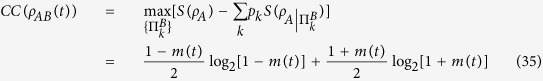


with 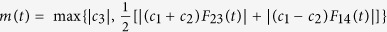. Finally, substituting Eqs (30) and (35) into


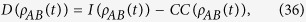


we can obtain the expression of quantum discord immediately.

## Additional Information

**How to cite this article**: Guo, J. L. and Zhang, X. Z. Quantum correlation dynamics subjected to critical spin environment with short-range anisotropic interaction. *Sci. Rep.* **6**, 32634; doi: 10.1038/srep32634 (2016).
